# The effects of mid- and long-term endurance exercise on heart angiogenesis and oxidative stress

**DOI:** 10.22038/IJBMS.2018.27211.6814

**Published:** 2018-08

**Authors:** Malihe Ardakanizade

**Affiliations:** 1 Department of Sport Sciences, Faculty of Humanities Damghan University, Semnan, Iran

**Keywords:** Angiopoietin-1, Endurance Exercise, HDAC4, MMP-2, VEGF-B

## Abstract

**Objective(s):**

Long-term, irregular endurance exercise may result in disturbance to the angiogenesis of heart muscles and blood supply. The aim of the present study is to evaluate the effects of mid- and long-term endurance exercise on the process of angiogenesis.

**Materials and Methods:**

Eighteen male Wister rats of 220±10 g, were randomly assigned to three groups of 6 rats including: Control, Mid, and Long Group. After the training sessions, the rats were weighed and sacrificed.

**Results:**

In comparison to the Control Group, the both groups, indicated remarkable increase in the weight of heart and significantly higher serum LDH and CK activity (*P*<0.01). In addition, after the training sessions, weakened antioxidant heart system (TAC, total thiol groups, and GPX activity) and increased oxidative stress markers (MDA and NO) were remarkably observed in Mid Group and particularly in those in the Long Group in comparison to the Control Group (*P*<0.05). Finally, significant increase in VEGF-B, MEF-2C and MMP-2 gene expression was found for both experimental groups, associated with the up-regulation of ANGPT-1 and HDAC4 in the Mid Group (*P*<0.05). While the longer exercise period induced significantly upper VEGF-B, MEF-2C, and MMP-2 and significantly lower ANGPT-1 and HDAC4 in the Long Group (*P*<0.05).

**Conclusion:**

In this study, higher oxidative status and upper angiogenic gene expression with higher VEGF-B, MEF-2c, and MMP-2 and lower ANGPT-1 and HDAC4 were traced as effects of long-term endurance exercise. These results point to the dis-regulation of blood supply in the presence of angiogenesis resulting from long-term exercise.

## Introduction

Regular exercise plays an important role in reducing one’s obesity-related problems and optimizing her lifestyle and cardiovascular health. It is well-recognized that regular exercise would prevent and treat several cardiovascular diseases, such as hypertension and coronary heart disease. Physical activity reduces the probability of cardiovascular mortality by 35% (1), resulting in an increase in life expectancy. Paradoxically, the potentially harmful effects of long-term intensive exercise on the risk of cardiovascular disease have been also highlighted and led to serious concerns (2, 3). On the other side of the coin, functional, electrical and structural remodeling of heart has been performed through studying long-term exercise in athletes. Research on hypertrophy, which increases the diameter of the left atrial, the thicknesses of the left ventricle wall, and the amount of cardiac mass, has shown that myocardial performance is affected by regular intensive physical activity (4). Vascular changes are also associated with neo-angiogenesis and capillary density in cardiac mass. Angiogenesis in heart muscles is a complex process mediated by the interaction of angiogenic and angiostatic factors. Vascular endothelial growth factor-B (VEGF-B) and angiopoietin 1 (ANGPT-1), are the main angiogenic factors which enhance the proliferation of endothelial cells and speed the vascular regrowth within the infarction area (5). There is evidence that, with help from the myocyte enhancer factor (MEF)-2 family, these genes would stimulate vasculogenesis and angiogenesis during the vascular development (6). Among the different types of mechanisms that regulate gene expression, the epigenetic changes associated with histone acetylation- deacetylation by various isoforms of histone deacetylase (HDAC) are also important (7). It is also found that changes in the oxidation/antioxidant balance would affect angiogenic activity in tissues (8). These oxidative changes result from reactive oxygen species (ROS). ROS are free-radical and non-radical compounds driven from oxygen, with the ability to modify the structure of proteins, lipids, carbohydrates, and nucleic acids. Multiple sources have attributed ROS mitochondrial leakage to radicals derived from xanthine oxidase by inflammatory cells. In the process of oxidation, on the other hand, the high rate of destruction of red blood cells resulting from intensive endurance exercise may increase free irons, which would contribute to protecting highly reactive hydroxyl radicals via iron-catalyzed Haber-Weiss reactions (9). 

Remodeling muscular heart tissues via strenuous and exhaustive physical activity is also reported in the literature (10). It is discussed that the changes exerted on these tissues are accompanied by the increased activity of various enzymes such as the matrix metalloproteinase (MMP). It is detected that, of different tissue proteases, MMP-2 deteriorates ECM components, affects tissue remodeling in pathological situations, and stimulates the process of angiogenesis (11, 12).   

Following the research trend reported above, researchers have shown great interest in revealing types of changes happening in heart muscles as a result of short and long-term endurance exercise and clarifying the molecular pattern of tissue remodeling. So, the present study was undertaken to elucidate the effects of the exercise pattern on oxidative changes and angiogenesis in heart muscles. 

## Materials and Methods

***Experimental design and exercise pattern***

In this study, eighteen male Wistar rats with an average weight between 210-230 g were sampled for the purpose of experimentation. The sample rats were kept in cages with a standard size (33 × 23×12 cm^3^) and under standard conditions with the temperature being about 25 ± 2 °C. Throughout the period of acclimatization and experiment, a standard lighting system, proper commercial rat chew diet, and tap water *ad libitum *were employed. These experiment conditions were in line with the codes issued by the Ethics Committee in Hamadan University of Medical Sciences and those issued by the National Medical Board, as “Principles of Laboratory Animal Care”. After one week of acclimatization, the rats were randomly divided into three groups of six rats; i.e., Control Group, Mid Group, and Long Group. The Control Group received no training, while the Mid Group underwent mid-term endurance exercise (1 hr swimming/day, 5 times/week, for 10 weeks) and the Long Group underwent long-term endurance exercise (1 hr swimming/day, 5 times/week, for 5 weeks). For the Long Group, exercise was gradually hardened up to 4.5 hr swimming/day, 5 times/week at the end of the tenth week (13).

***Sample collection***

The sample rats were sacrificed under aesthetic conditions, as approved by the ethics codes from “Principles of Laboratory Animal Care”. The body weight and length of the samples from mouth to tail were measured in order to analyze their body surface area (BSA) (14). The BSA was calculated according to the following formula: 

$\mathrm{BSA}=6.67\times W^{0.7}\times [\frac{0.34}{\frac{\sqrt[{3}]{W}}{L}}]$

Where W and L stand for body weight in grams and body length in centimeters, respectively. 

The blood samples were drawn via aspiration from the caudal vena cava and the blood plasma was taken via cenrifugatin at 5000×g for 10 min. The heart muscle was dissected and then rapidly washed using cold PBS and precisely weighed. Then, the left ventricle was dissected and weighed. Finally, the left ventricle was kept under freezing conditions at -80 °C for the purpose of examining its molecular and biochemical structure. 

***Myocardial oxidant analysis***

A sample of the frozen tissues (about 20 mg) was homogenized in the lysate buffer containing 1% Triton X-100, 500 mM, Tris/HCl, pH 7.6, 200 mM NaCl, and 10 mM CaCl_2_ by using a protease inhibitor cocktail [Sigma-Aldrich Co Ltd, Dorset, UK]. After 15 min of immersion of the homogenized tissues in the lysis buffer, the tissues were centrifuged for 5 min at 4°C and 14000×g and the supernatant was used to measure the protein level of the tissues through the bicinchoninic acid (BCA) method (15). Following the method described by Hu *et al.* and expressed as nmol.mg^-1^ of protein (16), the total thiol (SH) groups of protein were also measured using 5, 5′-dithionitrobenzoic acid (DTNB. The end product of myocardial muscle lipid peroxidation, malondialdehyde (MDA), was measured at 535 nm and 525 nm via thiobarbituric acid and n-butanol (17). and was expressed as pmol.mg^-1^ of protein. Following the method proposed by Benzi and Strain (18), total antioxidant capacity (TAC) was assessed based on the strength of the antioxidant solution power to reduce ferric ion (Fe3+) found in the 2,4,6-Tris(2-pyridyl)-s-triazine (TPTZ) solution to the ferrous ion (Fe2+). NO3−/NO2− and Glutathione peroxidase (GPX) activity were measured through the ZellBio colorimetric assay kit (Ulm-Germany) and the results were respectively expressed as U/µg protein and nmol/mg protein. 

***Real-Time PCR***

Extraction of RNA was performed via the TRizol (Invitrogen, Thermo Fisher Scientific, and USA). After quantification and qualification of the sample via the NanoDrop One^c^ UV-Vis Spectrophotometer (Thermo Fisher Scientific, USA), the extracted RNA was reversed to complementary DNA (c-DNA) by using the cDNA synthesis kit (Thermo Fisher Scientific, USA).Then, triplicate quantitative real-time PCR was carried out by using the SYBR *premix *Ampliqon (Odense M, Denmark) on a LightCycler® 96 System (Roche Life Science Deutschland GmbH Sandhofer, Germany) (19). The genes amplified in this stage are listed in Table 1 in which the genes are compared with hypoxanthine phosphoribosyltransferase 1 (HPRT1) as a housekeeping gene. The calculations reported were done through the comparative Ct method and fold change analysis based on the 2^−^^△△^^Ct^ formula (19). 

***Statistical analysis***

The descriptive statistics of the data collected in this study were expressed in terms of mean and standard deviation. On the other hand, the SPSS software, version 16, was used to run inferential statistics on the collected data. One-way analysis of variance (ANOVA), followed by the *post hoc* Tukey test, was performed to compare the results for the three groups of rats. The level of statistical significance for all the conducted analyses was set at *P*<0.05.

## Results

***Serum enzyme activity***

The serum enzyme activity of the control and trained groups is summarized in Table 2. According to the table, when compared with the rats in the Control Group, the rats in the Mid Group and Long Group indicated statistically significant increase in their creatine kinase (CK) activity (*P*=0.001) after the training programs designed. Also, after endurance exercise, lactate dehydrogenase activity showed significant increase for the rats in the Mid Group (*P*=0.025) and the Long Group (*P*=0.001) in comparison to the rats in the Control Group. Interestingly, the cardiovascular and muscular effects of long-term endurance exercise were very sizeable, as evidenced by the significantly higher the activity of these two enzymes for the Long Group than the Mid Group after the training sessions (*P*=0.001). 

***Heart weight relative to body area***

In this study, the heart weight, left ventricular weight, and body surface area of the samples were measured and, their heart mass and ventricular mass relative to their body surface were calculated. The results have been indicated in Table 3. The results show that the heart weight was significantly higher for the rats in both the Mid Group (*P*=0.002) and the Long group (*P*=0.001) in comparison to the rats in the Control Group once the training sessions on endurance exercise were held. In this study, the left ventricle weight was considered as an index of the diameter of the left ventricle and the diameter was not separately measured. With respect to the left ventricular weight after the training sessions, the weight was showed significant increase only for the rats in the Long Group, and not the Mid Group, when compared with the rats in the Control Group (*P*=0.001). Measurement of BSA, which is an index of the body surface relative to the body weight, was also undertaken. Monument indicated that, after the training sessions, the value of the BSA significantly decreased for the Long Group when compared with the Mid Group (*P*=0.005) and the Control Group (*P*=0.002). On the other side of the coin, the ratio of the heart weight to the BSA was significantly higher in both the Mid Group (*P*=0.004) and the Long Group (*P*=0.001) than the Control Group. Finally, the ratio of the left ventricle to the BSA was significantly higher in the Long Group in comparison to both the Mid Group and the Control Group (*P*=0.001) after the training sessions. 

***Oxidant/antioxidant parameters in all groups***

**Figure 1:**
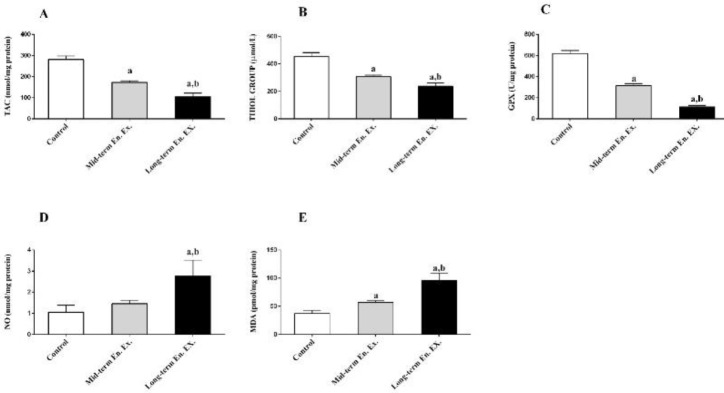
represents (A) the total antioxidant capacity (TAC), (B) the total thiol group (C) the glutathione peroxidase (GPX) activity, (D) the nitric oxide (NO) level, and (E) the malondialdehyde (MDA) level in the left ventricle muscle. The data are represented in terms of mean and SD. a, *P*<0.05 versus control group. b, *P*<0.05 in long term endurance exercise (long. End. Ex.) versus mid-term endurance exercise (Mid. End. Ex)

As depicted in Figures 1A, 1B and 1C, mid-term endurance exercise, induced oxidative stress in the heart muscles of the rats, as represented by the significant decrease in their total antioxidant capacity (TAC), total thiol groups and GPX activity (*P*=0.001). Moreover, in comparison to mid-term endurance exercise, long-term exercise induced significantly higher oxidative exercise in the rats , as represented by the lower heart TAC level (*P*=0.001), serum total thiol groups level (*P*=0.001) and heart GPX activity (p=0.001). Also, the oxidative radicals induced by ROS resulted in higher nitric oxide level (NO) in the rats trained on long-term endurance exercise in comparison to the rats receiving either mid-term exercise or not exercise at all (*P*=0.001), and the lipid peroxidation induced by malondialdehyde, significantly increased after mid, and long-term exercise (*P*=0.002 and *P*=0.001, respectively). Finally, in comparison to the rats undergoing a lower period of training, those trained on longer period indicated a higher MDA level (*P*=0.001). 

***Evaluation of gene expression***

**Figure 2:**
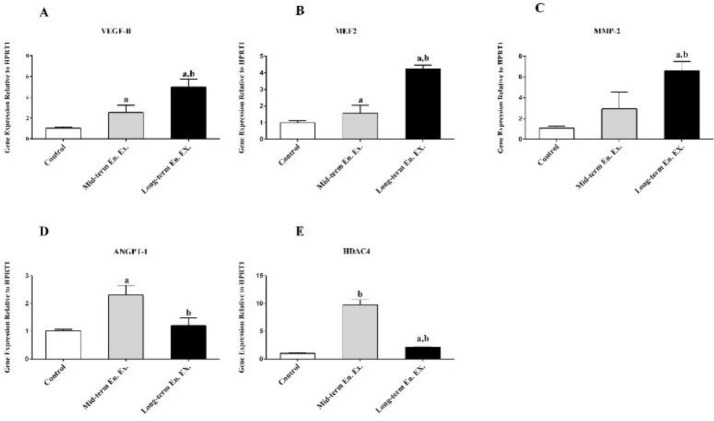
Represents the expression of (A) VEGF-B, (B) MEF-2, (C) MMP-2, (D) ANGPT-1, and (E) HDAC4 genes in the left ventricle muscle. The data are represented in terms of mean and SD. a, *P*<0.05 versus control group. b, *P*<0.05 in long term endurance exercise (long. End. Ex.) versus mid-term endurance exercise (Mid. End. Ex)

The schematic representation for the analysis of gene expression is depicted in Figure 2. The statistical analysis indicated that the rats in both experimental groups (Mid and Long Group) has higher up-regulation of the VEGF-B and MEF2C genes after the sessions on endurance exercise (*P*=0.001). Moreover, endurance exercise over a long period of time resulted in higher expression of the VEGF-B and MEF2C genes when compared with endurance exercise over a lower period of time (*P*=0.001) (Figures 2A and 2B). In a similar fashion, the expression of the MMP-2 gene significantly increased in the case of those rats trained over a long period of time (*P*=0.001) when compared with the rats trained over a lower period of time (Figure 2C). As showe in Figures 2D and 2E, in comparison to the Control Group, the expression of the ANGPT-1 and HDAC4 genes in the Mid Group was up-regulated significantly higher (*P*=0.001). Interestingly, unlike the ANGPT-1 gene, the expression of the HDAC4 gene was down-regulated in the case of the Long Group (*P*=0.001). 

## Discussions

The results of the present study indicated that alterations in skeletal muscles and considerable plasticity of cardiac and skeletal muscles occurred as a result of the sample rats performing endurance exercise. In this regard, it is argued that changes in the signaling cascade at the level of transcription and translation would lead to changes in muscle contractibility and consequently, cardiovascular capacity (20). The significant increase in the contractibility of heart muscles was accompanied by a significant increase in the weight of the heart and left ventricular. Furthermore, in this study, physical changes due to endurance exercise were accompanied by a decrease in the body volume as shown by the BSA analysis. In addition, the considerable changes in the muscles were accompanied by a significant increase in the creatine kinase and lactate dehydrogenase. These changes represent disturbance to metabolic muscles resulting from intensive endurance exercise (21, 22). The changes in the muscles and the disruption in the structure of the sarcolemma membrane would lead to the release of the CK into the bloodstream (23), which represents the severity, duration, and intensity of muscle soreness. Moreover, increase in the serum activity of the lactate dehydrogenase (LDH), i.e., the enzyme couples the inter conversion of the lactate and pyruvate with NADH and NAD^+^, represents the intensity and duration of the exercise program undertaken (24). Similarly, following the significantly higher level of the CK and LDH in the experimental groups after the exercise sessions as observed in the present study, it can be concluded that, in comparison to mid-term endurance exercise, endurance exercise over longer periods of time would induce more extreme disturbance to the heart and the muscle. In the literature, a similar increase in the levels of the serum LDH and CK after strenuous, prolonged training has been reported (25, 26).

**Table 1:** Detail of primers for Real-time PCR

Gene Name	Primer Sequence	Accession number	Base pair
HPRT1	Forward CCTCCTCAGACCGCTTTTCC Reverse CACTAATCACGACGCTGGGA	NM_012583.2	75
VEGF-B	Forward GCAACACCAAGTCCGAATG Reverse CTTCACAGCACTCTCCTTTCT	NM_053549	124
ANGPT-1	Forward ACAAAGGACGCTGATAACGAC Reverse AGTAGTGCCACTTTATCCCAT	NM_053546	157
MMP-2	Forward CCCCTATCTACACCTACACCA Reverse GCGATGCCATCAAAGACAATG	NM_031054	178
HDAC4	Forward GACAGAAACTGGACAGCTCG	NM_053449.1	135
	Reverse CCACTACACAGCCTACAGCC		
MEF2C	Forward CTGAGGATGTGGACTTGCTGT Reverse GCTGCTCAGAGAGTATTCGGTA	NM_006223957.3	170

**Table 2:** Serum enzyme activity in experimental and control groups

**Parameters**	**Control**	**Mid. End. Ex.**	**Long. End. Ex.**
LDH (U/L)	181.50 ± 57.12	242.83±6.17^a^	349.67±16.02^a^,^b^
CK (U/L)	53.67 ± 10.63	126.17±6.01^a^	155.33±13.29^a^,^b^

All the data are represented in term of mean and SD. In each row,

Mid. End. Exc. represents Mid-term endurance exercise, Long. End. Ex. represents Long-term endurance exercise

a versus control group,

b Long. End. Ex. versus Mid. END. Exc. *P*<0.05 represents a significant difference between the groups.

**Table 3:** Serum enzyme activity in experimental and control groups

**Parameters**	**Control**	**Mid. End. Ex.**	**Long. End. Ex.**
Heart Weight	1.10 ± 0.02	1.15±0.01^a^	1.22±0.01^a^,^b^
Left Ventricular Weight	0.72 ± 0.00	0.73±0.01	0.81±0.01^a^,^b^
BSA (cm2)	419.47 ± 5.94	417.95 ± 3.29	407.67 ± 4.43^a^,^b^
Heart Weight/BSA (g/m2)	26.38 ± 0.63	27.67 ± 0.53^a^,^b^	29.97 ± 0.58^a^,^b^
Left Ventricular Weight/BSA (g/m2)	17.12 ± 0.39	17.50 ± 0.38	20.03 ± 0.34^a^,^b^

All the data are represented in terms of mean and SD. In each row,

Mid. End. Exc. represents Mid-term endurance exercise, Long. End. Ex. represents Long-term endurance exercise, and BSA, stands for Body surface area

a versus control group,

b Long. End. Ex. versus Mid. END. Exc. *P*<0.05 represents a significant difference between the groups.

In this study, the disturbance to the muscles was also accompanied by oxidative changes. In fact, the results indicated that oxidative stress is associated with endurance exercise over variable periods of time. The oxidative changes observed in the present study, which happened in were with the increase in the oxidative markers of the nitric oxide (NO) and lipid peroxidation end product (MDA), are attributed to the considerable decrease in the TAC, and serum thiol level. Similarly, based on the results of the study, it is discussed that endurance exercise, is accompanied by mitochondrial ROS generation include hydroxyl, peroxyl, superoxide radicals and nitrogen species as nitric oxide (27). The ROS produces hydroperoxides that are worn off by the GPx containing reduced thiol. Thus, stemming from long-term detoxification of hydroperoxides, it is discussed that increase in the ROS level, as observed in the present study, would lead to decrease in the GPX activity and reduced thiol groups. On the other hand, the decrease in the antioxidant capacity was more representative of longer-term training, pointing to the increase in the oxidative changes exerted by the duration of the exercise program. As detoxification is limited by the antioxidant system, the excess of the ROS, the existence of attack fatty acids, and the degeneration of fatty oxides point to the MDA production. This mechanism was observed in the present study. Similarly, oxidative stress resulting from strenuous exercise has been already reported in the field (28, 29). The generation of nitric oxides serves a function from the function served by other oxidative products. In long-term endurance exercise, vascular expansion occurs due to the need of skeletal and cardiac muscles for blood supply. Therefore, the NO is implicated in the blood supply to help the arteries of both coronary and skeletal muscles via the endothelial nitric oxide synthase (eNOS) (30). The provision of this blood supply is necessarily accompanied by an increase in NOs and the expression of the vascular endothelial growth factor beta (VEGF-B) gene in the molecular pattern. The VEGF-B gene is up-regulated through the interaction of the hypoxia inducible factor - 1 (HIF-1) and hypoxia responsive element (HRE), as a response to the hypoxia induced by long-term endurance exercise (31, 32). The VEGF-B, gene stimulates proliferation of endothelial cells and migration and formation of new vessels through signal transduction done by tyrosine kinase receptors (33). Thus, the need for coronary and skeletal muscles for blood supply is met by the process of angiogenesis and the VEGE-B gene expression. As the expression of the VEGE-B gene went higher in the Long Group as a result of long-term endurance exercise, a higher level of angiogenesis was traced in this group. In addition, production of the ROS would stimulate the expression of pro-angiogenic growth factors, like the VEGF-B gene (34), as evidenced in this study. Thus, higher production of the ROS, which occurred in concomitant with the higher expression of the VEGF-B gene, would confirm the relationship between angiogenesis and oxidative stress. 

Exercise induced calcineurin signaling pathways in the muscles, in order to provide the process of muscle contraction with calcium needed. In the literature, it has been showen that regulation of calcineurin-dependent genes, is mediated by MEF2 transcription factors (35). On the other hand, it has been also indicated that the gene expression of MEF-2c can be up-regulated in endothelial cells through the VEGF-B, which in return, regulates the process of vasculogenesis (6). Therefore, the considerably higher expression of the VEGF-B gene, along-with the VEGF-β gene, which was effected by endurance exercise represents an increase in the contractibility and vascularization of heart muscles. Angiopoietin 1 (ANGPT-1) is the protein supporting the survival of endothelial cells through the process of angiogenesis (36). The vascular permeability induced by the VEGF-B can be blocked and regulated through the overexpression of the ANGPT-1 gene (37). In this way, the density of the new vessels can be adjusted. Accordingly, the higher expression of the ANGPT-1 gene resulting from mid-term endurance exercise could contribute to the regulation of angiogenesis, a case not seen with respect to long-term endurance exercise in this study. Thus, it could be concluded that, in long-term endurance exercise, the dis-regulation and formation of new blood vessels were accorded. Recently, it has been reported that the concomitant expression of VEGF-B and angiopoietin-2 would increase and regulate micro vessel density and permeability (12).

The matrix metalloproteinase-2 (MMP-2) is a zinc-dependent protease that plays an important role in regulation of extracellular matrix and heart angiogenesis. It is reported that the angiogenic activity of the MMP-2 leads to its collagenolytic activity, which in return, leads to an increase in endothelial cells in order to invade the basement membrane of the vascular endothelium (38). Thus, the effect of the higher expression of MMP-2, along with VEGF-β, on the process of angiogenesis was an observation that is in line with previous research findings about angiogenesis. The epigenic mechanism controlling the expression of gene result from both histone acetylases and histone deacetylases (HDACs) (39). While it is reported that the down-regulation of HDAC4 increases the expression of the VEGF-B gene and angiogenesis (40), the higher expression of the HIF-VEGF signaling gene through the phosphorylation of the HDAC4 protein in the cerebral ischemia has been also reported (41). In the present study, the opposite expression of these two genes was observed. It has been reported that the down-regulation of VEGF-B mediated by HDAC4 is effected through inhibition of the runt-related transcription factor2 (Runx2). It has been also indicated that over-expression of the Runx2 would induce VEGF-B mRNA and protein expression under both normoxic and hypoxic conditions (42). Thus, the deacetylation of the Runx2 decreases its expression and down-regulates the expression of VEGF-B (42). Our result is in line with the previous findings about the role of HDAC4 in the process of heart angiogenesis. Accordingly, the significantly higher expression of the HDAC4 gene was observed as accompanied by the lower expression of the VEGF-B gene. Similarly, as the expression of the HDAC4 gene was down-regulated, the expression of the VEGF-B gene was heightened. 

## Conclusion

In general, the results of the present study showed that increasing the duration of endurance exercise, would induce considerable higher serum activity of the CK and LDH. This refers to the deleterious effects of over-training on skeletal and cardiac muscles. In addition, significant oxidative stress was traced in this study, which resulted from longer periods of endurance swimming. This significantly higher oxidative stress was accompanied by the increase in the rate of angiogenesis. Moreover, the dis-regulation of the vessel permeability was accompanied by the higher expression of the VEGF-B and lower ANGPT genes. Increased contractibility of the heart muscle resulting from long-time endurance exercise was too accompanied by an increase in the weight of the heart and left ventricle on one side and the up-expression of the MEF-2C gene on the other side. Furthermore, in this study, an increase in the expression of MMP-2 genes was observed to stimulate VEGF-B. The epigenic regulation of VEGF-B gene was also accompanied by deacetylation and down-regulation of its inhibitor via HDAC4. At the end, it can be concluded that long term endurance exercise has deleterious dis-regulative effects on the angiogenesis of heart muscle and blood supply. 
